# Comparative *in vitro* assessment of CYP2C19 inhibition by ilaprazole and conventional proton pump inhibitors using a high throughput fluorometric assay

**DOI:** 10.1038/s41598-025-02872-5

**Published:** 2025-05-25

**Authors:** A. Priyadharshini, T. M. Vijayakumar, N. Damodharan, K. M. Vasanth, Arun Elaiyaraja

**Affiliations:** 1 Department of Pharmacy Practice, SRM College of Pharmacy, SRM Institute of Science and Technology, Kattankulathur, Chengalpattu District, Tamil Nadu 603203 India; 2 Department of Pharmaceutics, SRM College of Pharmacy, SRM Institute of Science and Technology, Kattankulathur, Chengalpattu District, Tamil Nadu 603203 India; 3 Department of Clinical Pharmacology, SRM Medical College Hospital and Research Centre, SRM Institute of Science and Technology, Kattankulathur, Chengalpattu District, Tamil Nadu 603203 India; 4 Drug and Poison Centre ( A Unit of Department of Pharmacy Practice), SRM Medical College Hospital and Research Centre, SRM Institute of Science and Technology, Kattankulathur, Chengalpattu District, Tamil Nadu 603203 India

**Keywords:** Proton pump inhibitors, Ilaprazole, Invitro study, CYP2C19 inhibition, Drug discovery, Medical research

## Abstract

Proton pump inhibitors (PPIs) are commonly used anti-ulcer agents, known to inhibit CYP2C19, leading to pharmacokinetic drug-drug interactions (DDIs). Ilaprazole is a newer PPI with a distinct pharmacokinetic profile that is predicted to overcome CYP2C19 inhibition. The current study aimed to predict the CYP2C19 inhibitory potential of Ilaprazole versus conventional PPIs (Omeprazole, Lansoprazole, Pantoprazole, and Rabeprazole) on CYP2C19 activity using a high-throughput fluorometric assay. The Vivid™ CYP2C19 Blue Screening Kit was utilized, and fluorescence intensity was measured after incubation with PPIs, using ticlopidine as a positive inhibitor control. The inhibition percentage was calculated, and IC50 values were determined using nonlinear regression analysis in graph pad prism, further, as per regulatory guidance, the C_max,u_/K_i_,_u_ ratio was assessed to interpret the potential for clinical drug-drug interactions of PPIs. The concentration-dependent inhibition of CYP2C19 activity by all PPIs was evaluated. The order of inhibition potency, based on IC50 and K_i_,_u_ values, was found to be: Omeprazole > Lansoprazole > Pantoprazole > Rabeprazole > Ilaprazole. The results of the C_max,u_/K_i_,_u_ ratio indicate that Omeprazole (0.0288) exceeded the cut-off value consistent with its well-documented *in vivo* CYP2C19 inhibitory effect. While Lansoprazole (0.00332) had a relatively higher ratio than Ilaprazole (0.00224), Pantoprazole (0.00124), and Rabeprazole (0.000635), all values remained below the regulatory cutoff, indicating minimal inhibition risk. Omeprazole is the most potent CYP2C19 inhibitor, as it exceeded the regulatory threshold guidelines for *in vitro* study, while other tested PPIs, including Ilaprazole, did not meet this cutoff, suggesting a lower likelihood of clinically significant inhibition. Although previous in vivo studies suggest variable inhibition with other PPIs, current data support the need for further head-to-head in vivo comparisons, particularly between Pantoprazole, Rabeprazole, and Ilaprazole, to determine the most suitable option in clinical scenarios involving CYP2C19 substrate.

## Introduction

In drug discovery, the assessment of pharmacokinetic drug interactions with the cytochrome P450 (CYP450) enzyme family is crucial. The CYP450 family consists of multiple isoforms (e.g., CYP3A4, CYP2C19, CYP2D6), which play an important role in the oxidative metabolism of several drugs. Inhibition of their activity (CYP450 isozymes) can lead to significant drug-drug interactions, affecting the clinical efficacy of co-administered medications or leading to toxicity^1,2^.

Generally, inhibition of CYP450 enzymes, particularly CYP2C19, can change the pharmacokinetics of many medications, it is of great clinical importance^3^. CYP2C19 is the major enzyme in the metabolism of PPIs, such as Omeprazole, Esomeprazole, Lansoprazole, and Pantoprazole. CYP2C19 inhibition by certain PPIs, including omeprazole, esomeprazole, pantoprazole, and lansoprazole, can lead to clinically relevant drug-drug interactions (DDIs). For example, the co-administration of clopidogrel with omeprazole or esomeprazole reduces clopidogrel’s efficacy, increasing the risk of thrombotic events^4,5^. Similarly, omeprazole reported significant interactions including affecting the diazepam metabolism and increased warfarin levels due to CYP2C19 inhibition^6,7^.

Ilaprazole, a novel proton-pump inhibitor was reported to be efficacious, tolerable, and safe, compared with the conventional PPIs in the management of both gastric and duodenal ulcers. At variance with other PPIs, ilaprazole was primarily metabolized by CYP3A4. Hence the literature suggested that the pharmacokinetics (PK) and pharmacodynamics (PD) of ilaprazole were not influenced significantly by CYP2C19 polymorphism and it also exhibits minimal CYP2C19 inhibition when compared to conventional PPIs^8–10^.

Fluorometric assays are sensitive, high-throughput, and cost-effective use in the early stages of drug development as they assess the enzyme activity and inhibition in real-time. CYP450 inhibition fluorometric assays function by employing specific substrates that are metabolized by a particular CYP450 isoform to produce fluorescent products. The quantification of inhibition is made possible by the drop in fluorescence caused by a decrease in substrate metabolism when an inhibitor is present^11,13^.

These inhibition investigations can be carried out on a dependable platform like Vivid® CYP450 Screening Kits which contains Recombinant human CYP450 enzymes, fluorescent substrates, and a regeneration system to maintain enzyme activity. These Screening Kits facilitate the measurement of interactions between drug and CYP450 enzymes using a simple “mix-and-read” fluorescent assay that is designed for high-throughput screening in multi-well plates. It helps in identifying the compound-CYP enzyme interactions, thereby unsuitable compounds can be eliminated in the drug discovery process. The current study aimed to predict CYP2C19 inhibitory potential compared with other proton pump inhibitors using the fluorometric assay^14,15^.

## Materials and methods

### Materials

Black-walled, clear-bottom 96 well microplates (Corning; Costar #3915).

Vivid™ CYP2C19 Blue Screening Kit (Thermo Scientific; Cat. No. P2864).

Vivid® CYP450 2X Reaction Buffer 100 Mm, CYP2C19 BACULOSOMES® Plus Reagent 0.5 nmol (Recombinant human cytochrome P450), 0.5 mL Vivid® Regeneration System (Glucose-6-phosphate and 30U/mL Glucose-6-phosphate dehydrogenase in 100 mM Potassium phosphate), 0.5 mL Vivid® NADP + , Vivid® EOMCC Substrate 0.1 mg, Vivid® Blue Fluorescent Standard 0.1 µmol.

Drugs (API):

Rabeprazole & Pantoprazole; Omeprazole, Lansoprazole & Ilaprazole (Tokyo Chemical Industry, India), Ticlopidine (Sigma-Aldrich, USA). (Purchased).

### Preparation of reagents

- 1X Vivid® CYP450 Reaction Buffer: Diluted 10 mL of 2X buffer with 10 mL nanopure water.

Fluorescent standard:

- Reconstituted 0.1 µmol standard with 1 mL DMSO (100 µM stock).
- Prepared 500 nM standard (5 µL of stock + 995 µL 1X buffer).

Serial dilution:

- 500 nM standard (200 µL) in wells A1/A2, two-fold dilutions down to H1/H2.
- Blanks: H1/H2 (1X buffer only).

### Preparation of solutions

Test compounds: Dissolved in DMSO; diluted to 2.5X in 1X buffer.

Positive control inhibitor (Ticlopidine): Stock in DMSO; 2.5X dilution in 1X buffer.

Master Pre-Mix: P450 BACULOSOMES® (50 µL) + Vivid® Regeneration System (100 µL) in 1X buffer.

Substrate/NADP + Mixture: Reconstituted substrate (0.1 mg in 205 µL acetonitrile); added 50 µL substrate + 30 µL NADP + (100X).

### High throughput fluorometric assay method

A high-throughput fluorometric assay was conducted using a black 96-well microplate. Fluorescence measurements were recorded on a BioTek Synergy H1 multimode microplate reader (Agilent, US) with the appropriate excitation/emission wavelength. The assay followed the protocol provided by Life Technologies, USA. Test compounds were evaluated based on their ability to inhibit fluorescent signal production in reactions involving recombinant CYP isozymes and specific substrates. For IC50 determination, two-fold serial dilutions of the test samples were prepared, and the plates were incubated at 37 °C for 20 min. The enzymatic reaction was initiated by adding a mixture of NADP + and the appropriate substrate, followed by a 10-min incubation at 37 °C. The reaction was then stopped by adding 0.5 M Tris buffer (end-point assay). Fluorescence data was collected at eight different concentrations for both inhibitors and test compounds, with all measurements performed in duplicate.

### Analysis of results

Fluorescence data were collected after the incubation period, to calculate the percentage inhibition using the following formula:

$$\% \;{\text{Inhibition}} = \left( {{1} - \left( {{\text{X}} - {\text{B}}} \right)/\left( {{\text{A}} - {\text{B}}} \right)} \right) \times {1}00\%$$

X = Fluorescence intensity of test compound (PPIs), A = Fluorescence intensity of solvent control (Fluorescence standard), B = Fluorescence intensity of the positive inhibitor (Ticlopidine).

#### Determination of IC₅₀ and IC₅₀,_u_ (unbound IC₅₀)

IC50 values for CYP2C19 (the concentration required to inhibit 50% of CYP2C19 activity) inhibition were calculated using nonlinear regression analysis in GraphPad Prism 9 software.

IC₅₀,_u_ (unbound IC₅₀) was calculated using the formula:

$$\begin{aligned} {\text{IC5}}0,{\text{u}} & = {\text{IC5}}0/{\text{fu}},{\text{inc}} \\ {\text{IC}}_{{{50}}} & = {\text{Total}}\;{\text{IC}}_{50} \;{\text{value}}\left( {{\text{in}}\;\upmu {\text{M}}} \right) \\ \end{aligned}$$

fu, inc = Fraction unbound in the incubation system (0.02, as per regulatory assumptions for highly protein-bound drugs).

#### Determination of Ki,u

The unbound inhibition constant (K_i_,_u_) was determined using the Cheng-Prusoff equation:

$${\text{Ki}},{\text{u}} = {\text{IC5}}0,{\text{u}}/{1} + \left( {{\text{S}}/{\text{Km}}} \right)$$

S = Substrate concentration used in the assay, IC₅₀,_u_ = unbound half-maximal inhibitory concentration, K_m_ = Michaelis constant for the substrate.

Since the assay conditions involved substrate concentrations significantly lower than K_m_, K_i_,_u_ was approximated as IC₅₀,_u_/2, following regulatory recommendations.

#### Determination of C_max,u_ and C_max,u_/K_i_,_u_ Ratio

The Cmax values for Pantoprazole, Rabeprazole, Lansoprazole, and Omeprazole were obtained from reliable sources, primarily the highest recommended doses listed in the FDA drug labels^15–19^. However, for Ilaprazole, as no literature data was available, its Cmax was obtained from available pharmacokinetic studies

$$C_{max} ,u = C_{max} \times fu$$

C_max,u_ = unbound peak plasma concentration, Cmax = Maximum plasma concentration (from literature), fu = fraction unbound in plasma (0.01 as per the guidance for high protein-bound drugs).

To predict the clinical significance of CYP2C19 inhibition, the C_max,u_/K_i_,_u_ ratio was calculated.

As per FDA regulatory guidelines, if C_max,u_/ K_i_,_u_ value ≥ 0.02 suggests a significant potential for *in vivo* CYP2C19 inhibition. This threshold was applied to assess the potential for drug-drug interactions (DDIs)^20^.

## Results and discussion

Fluorometric assay is the most commonly used method to identify the CYP inhibition of compounds in early drug discovery due to its sensitivity, speed, low cost, and ease of use. This method helps to ensure the drug interaction potential of the test compound on the important CYP isoforms such as CYP2C19. Inhibitor potency was quantified by determining IC50 values, which in turn was carried out using fixed substrate and inhibitor concentrations. Ilaprazole, Conventional PPIs, and Positive Control (Ticlopidine) were assayed between concentrations ranging from 0.8 to 6.4 μg/mL (Eight Different Concentration). All samples were assayed in duplicate as per the experimental condition (Table 1), the endpoint mode was selected to analyze the percentage inhibition, and IC50 values were calculated.

**Table 1:** Experimental conditions for CYP2C19 fluorometric inhibition assay setup.

Assay setup	Experimental condition
Master pre-mix addition	50 µL
Vivid® substrate and Vivid® NADP + mixture	10 µL
Incubation time	30 min
Stopping reagent	50 µL–0.5 M Tris buffer
Fluorescence reader	Multimode microplate reader (Hybrid)
Excitation wavelength	415 nm (Band width:20 nm)
Emission wavelength	460 nm (Band width:20 nm)
Assay mode	Endpoint assay mode

**Fig. 1:**
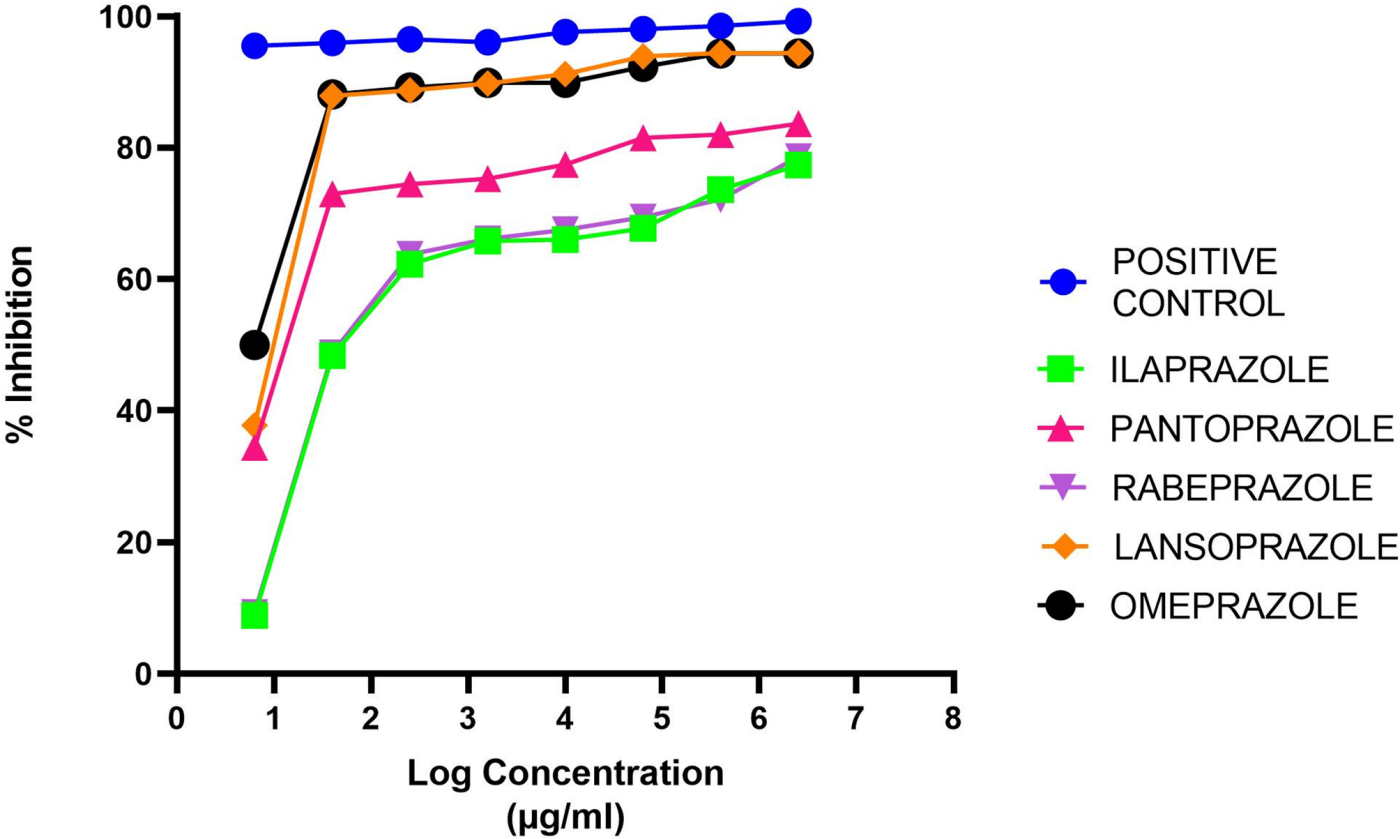
Concentration-dependent CYP2C19 inhibitory effect of Ilaprazole versus conventional PPIs, compared using ticlopidine as a positive inhibitory control.

This graph illustrates the inhibitory effects of varying concentrations of Ilaprazole and conventional PPIs (Omeprazole, Pantoprazole, Rabeprazole, and Lansoprazole) on CYP2C19 enzyme activity, with Ticlopidine serving as the positive control. The percent inhibition is plotted against the concentration (µL) to assess the relative potency of each compound in inhibiting CYP2C19 (Fig. 1).

Concentration-dependent % inhibition of the test compounds and positive control on CYP2C19 isozymes were observed. This concentration range showed good linearity of concentration-dependent percentage inhibition on CYP2C19.

The study findings indicate that Omeprazole exhibited the strongest CYP2C19 inhibition among the tested PPIs, with the lowest IC50 value (1.41 µM), suggesting its potent inhibitory effect even at lower concentrations. Lansoprazole followed closely (IC50 = 1.65 µM), demonstrating a relatively high inhibitory potential. Pantoprazole showed moderate inhibition (IC50 = 3.52 µM), whereas Rabeprazole (IC50 = 6.43 µM) and Ilaprazole (IC50 = 6.62 µM) exhibited the weakest inhibition.

The unbound inhibition constant (K_i_,u) further confirms these trends, with Omeprazole (K_i_,u = 35.25 µM) showing the strongest inhibition, followed by Lansoprazole (41.25 µM) and Pantoprazole (88 µM). In contrast, Rabeprazole (160.75 µM) and Ilaprazole (165.5 µM) displayed the weakest inhibition (Table 2). Based on IC50 and K_i_,u values, the inhibition potency ranked as Omeprazole > Lansoprazole > Pantoprazole > Rabeprazole > Ilaprazole^21^ This ranking differs from the findings of Li et al. (2004), where Rabeprazole exhibited greater inhibition than Pantoprazole^22^ This discrepancy may be attributed to differences in experimental conditions, enzyme sources, or drug concentrations.

**Table 2:** Comparative Inhibition of CYP2C19 by Ilaprazole and Conventional PPIs: IC₅₀, K_i_, and Cmax,u/K_i_,u Ratio Analysis.

DRUG	IC50 (µM)	IC₅₀,u (µM)	Ki,u (µM)	Cmax,u (µg/mL)	Cmax,u (µM)	Cmax,u/Ki,u
Ilaprazole	6.62 ± 1.34	331	165.5	0.0045	0.0123	0.00224
Pantoprazole	3.52 ± 1.47	176	88	0.025	0.0652	0.00124
Rabeprazole	6.43 ± 1.09	321.5	160.75	0.0062	0.0173	0.000635
Lansoprazole	1.65 ± 1.37	82.5	41.25	0.0115	0.0311	0.00332
Omeprazole	1.41 ± 0.96	70.5	35.25	0.0112	0.0324	0.0288

Following the determination of IC50 and K_i_,u, the C_max,u_ (Unbound Maximum Plasma Concentration) and C_max,u_/K_i_,_u_ ratio were calculated to assess the potential of CYP2C19 inhibition. According to current regulatory guidelines (ICH M12 & FDA), a C_max,u_/K_i_,_u_ ratio below 0.02 suggests that the risk of reversible inhibition can be reasonably excluded. In this study, only omeprazole (0.0288) exceeded this threshold, consistent with its well-documented *in vivo* CYP2C19 inhibitory effect. While Lansoprazole (0.00332) had a relatively higher ratio than Ilaprazole (0.00224), Pantoprazole (0.00124), and Rabeprazole (0.000635), all values remained below the regulatory cutoff, indicating minimal inhibition risk.

In summary, Omeprazole demonstrated the strongest inhibition of CYP2C19 in vitro, followed by Lansoprazole, Pantoprazole, and Rabeprazole, with Ilaprazole exhibiting the weakest inhibitory effect based on IC₅₀ and K_i_,u values. These findings suggest that Ilaprazole may pose the lowest risk for CYP2C19-mediated drug-drug interactions. Clinical studies support this, showing that Ilaprazole did not significantly alter Clopidogrel’s pharmacodynamic response in healthy volunteers and did not contribute to Clopidogrel resistance in acute stroke patients^10,23^.

However, according to current regulatory thresholds (ICH M12 and FDA guidance), only omeprazole exceeded the C_max,u_/K_i_,_u_ cutoff value of ≥ 0.02, which indicates that the potential for clinically relevant CYP2C19 inhibition cannot be excluded. None of the other tested PPIs, including ilaprazole, crossed this threshold, suggesting a lower risk of *in vivo* inhibition. Notably, previous *in vivo* evidence has reported CYP2C19-related interactions with PPIs such as Lansoprazole and Pantoprazole, suggesting variability based on patient populations and clinical settings^4,24^. Therefore, although Ilaprazole shows favorable in vitro and clinical profiles, further head-to-head *in vivo *studies, particularly among Pantoprazole, Rabeprazole, and Ilaprazole, are essential to validate these findings and determine the most appropriate PPI with minimal CYP2C19-mediated interaction risk.

## Conclusion

This study demonstrates that among the tested PPIs, Omeprazole is the most potent CYP2C19 inhibitor, as it exceeded the regulatory threshold guidelines for* in vitro* study, while other tested PPIs, including Ilaprazole, did not meet this cutoff, suggesting a lower likelihood of clinically significant inhibition. Among these, Ilaprazole consistently exhibited the weakest inhibitory effect based on IC₅₀, K_i_,_u_, values, indicating a comparatively safer profile regarding CYP2C19-related drug interactions. Although previous in vivo studies suggest variable inhibition with other PPIs, current data support the need for further head-to-head *in vivo* comparisons, particularly between Pantoprazole, Rabeprazole, and Ilaprazole, to determine the most suitable option in clinical scenarios involving CYP2C19 substrate.

## Data Availability

The datasets used and/or analysed during the current study available from the corresponding author on reasonable request.
